# Association of Inflammation with Metabolic Syndrome among Low-Income Rural Kazakh and Uyghur Adults in Far Western China

**DOI:** 10.1155/2015/706768

**Published:** 2015-07-01

**Authors:** Yi-Zhong Yan, Ru-Lin Ma, Yu-Song Ding, Heng Guo, Jing-Yu Zhang, La-Ti Mu, Mei Zhang, Jia-Ming Liu, Dong-Sheng Rui, Jia He, Feng Sun, Kui Wang, Shu-Xia Guo

**Affiliations:** ^1^Department of Preventive Medicine, University of Shihezi, Shihezi 832000, China; ^2^Department of Pathology and Key Laboratory of Xinjiang Endemic and Ethnic Diseases (Ministry of Education), Shihezi University School of Medicine, Shihezi 832000, China; ^3^Department of Epidemiology and Bio-Statistics, School of Public Health, Peking University Health Science Center, Beijing 100000, China

## Abstract

This study focused on low-income rural and nomadic minority people residing in China's far west and investigated their relationship between inflammatory markers (IL-6, hsCRP, FFA, and adiponectin) and MS and ethnic differences. And it found that improving behavioral lifestyle by education or using drugs to control inflammation may prevent MS. These observations may benefit low-income populations.

## 1. Introduction

Metabolic syndrome (MS) comprises a cluster of clinical metabolic diseases that include hypertension, insulin resistance (IR), obesity, and dyslipidemia [1–3]. MS is a global public s1/4–1/3 of the global population is affected by MS and that its prevalence will continue to increase [4].

China is a multiethnic country, with more than 10 ethnic groups in Xinjiang. In this region, Kazakh and Uyghur populations constitute large minority groups, with most individuals residing in low-income rural communities [5]. For example, more than 92% of Uyghurs in Jiashi County live on US $1.00 per day or less, and this percentage is much higher than the national average reported in 2005 (15.9%) [6, 7]. Due to a primitive economic system, limited public health resources, and a poor transportation system, few serious investigations have focused on the analyses of local public health issues, including the prevalence of hypertension, obesity, dyslipidemia, and related diseases such as diabetes and cardiovascular diseases (CVDs). Our previous studies demonstrated an MS prevalence of 21.2% in Uyghurs [8] and 26.6% in Kazakhs [9]; these values are significantly higher than the national average of 16.5% [10]. Differences in religion, culture, lifestyle, diet, and genetic background in these ethnic groups may be related to this high MS prevalence, and knowledge about these differences may be useful for establishing appropriate preventive public health policies for Xinjiang residents.

MS is a chronic, low-grade, systemic inflammatory state [11], and the relationship between MS and inflammation has long been acknowledged. Indeed, studies have demonstrated that inflammatory markers such as IL-6, hsCRP, FFA, and adiponectin play an important role in MS development and are closely related to the occurrence of MS and its components [12, 13]. IL-6 and CRP may contribute to MS risk [14], and a sharp increase in FFA can cause both insulin resistance (IR) in the liver, increase the expression of proinflammatory cytokines such as IL-6, and stimulate the liver to secrete CRP [15]. Decreased adiponectin levels are also associated with IR and are observed in proinflammatory states [16, 17].

However, these findings were primarily obtained in high-income and urban settings, whereas little information has been gathered in low-income rural settings. Specifically, data concerning inflammatory marker levels and their relationship with MS in Uyghur and Kazakh people and data concerning differences between these two local minority groups are lacking. In this study, we analyzed the relationships between inflammation and MS and the possible differences between these ethnic groups residing in far western China to examine the reasons underlying the incidence of MS in Xinjiang.

## 2. Materials and Methods

### 2.1. Ethics Statement

The Institutional Ethics Review Board (IERB) at the First Affiliated Hospital of the Shihezi University School of Medicine approved the study (IERB no. SHZ2010LL01). Standard university hospital guidelines, including informed consent, voluntary participation, confidentiality, and anonymity, were followed. All of the participants provided written informed consent before the study began.

### 2.2. Settings and Participants

This study was conducted from 2009 to 2012 among Uyghurs residing along the Bazi Xiang River region of Jiashi and Kazakhs residing in Nalati township of Yili in Xinjiang. For the basic survey, we divided subjects from the two ethic groups into MS and non-MS groups according to the 2005 IDF criteria. Using a random number table in SPSS 19.0, we then randomly selected 218 and 156 cases from the MS groups and 201 and 180 cases from the non-MS groups of the Uyghur and Kazakh populations, respectively, for laboratory testing.

### 2.3. Definition of MS and HOMA-IR

(1) MS was defined by central obesity according to the IDF (modified guidelines of the WHO for the Asia Pacific region) [18], a waist circumference ≥ 90 cm in men or ≥80 cm in women, plus any two of the following four factors: (a) an elevated triglyceride level of >150 mg/dL (1.69 mM); (b) a reduced HDL cholesterol level of <40 mg/dL (1.04 mM) in males or <50 mg/dL (1.29 mM) in females; (c) elevated BP (SBP ≥ 130 or DBP ≥ 85 mmHg); and (d) an elevated fasting plasma glucose level of ≥100 mg/dL.

(2) The homeostasis model assessment of insulin resistance (HOMA-IR) index was defined as follows: fasting insulin (in microinternational units [*μ*IU] per mL) × fasting glucose (in mM)/22.5 [19]. The Chinese Diabetes Society (CDS) states that IR can be estimated using this formula in epidemiological or clinical studies, and the upper quartile of the subjects was the split point for this study: 1.17 in Uyghurs and 1.23 in Kazakhs.

### 2.4. Exclusion Criteria

The exclusion criteria were as follows: (1) patients with serious heart and liver dysfunction; (2) patients using insulin and oral hypoglycemic, antihypertensive, lipid-lowering drugs; (3) pregnant women; (4) patients with cancer or tuberculosis or other infectious diseases.

### 2.5. Laboratory Tests

(1) Total cholesterol (TC), triglyceride (TG), low-density lipoprotein cholesterol (LDL-C), high-density lipoprotein cholesterol (HDL-C), and fasting glucose levels were assessed using a biochemical autoanalyzer (Olympus AU 2700, Olympus Diagnostics, Hamburg, Germany) in a clinical laboratory.

(2) IL-6 and adiponectin levels were determined by ELISA with kits purchased from Shanghai Westang Bio-Tech Co., Ltd. (Shanghai, China). hsCRP was determined by immunonephelometry, and FFA was determined by a colorimetric assay; the kits were purchased from Randox Laboratories Ltd. (UK). Insulin levels were determined by radioimmunoassay using a kit purchased from Beijing Atomic-Tech Co., Ltd. (Beijing, China).

### 2.6. Statistical Analysis

All of the analyses were performed using the SPSS statistical package for Windows (version 19.0). Continuously and normally distributed variables were analyzed using *t*-tests, and the results are presented as the means ± standard deviations (M ± SD); variables with a skewed distribution were analyzed using the Mann-Whitney *U*-test, and the results are expressed as the median (upper quartile, lower quartile) [M(Q_u_, Q_L_)]. All of the rates were compared using the Chi-square test. Differences of *P* < 0.05 were considered to be statistically significant.

## 3. Results

### 3.1. Description of the General Situation in the Uyghur and Kazakh Populations

Average age and gender were not significantly different between the MS and non-MS groups in the Uyghur and Kazakh populations or between the two ethnicities (*P* > 0.05 for each comparison). Among Uyghurs and Kazakhs, BMI, WC, SBP, DBP, TG, HDL-C, FPG, LDL-C, and TC levels were higher in the MS group than in the non-MS group (*P* < 0.05 for each comparison). However, there were no differences between the two ethnicities, regardless of whether they were classified in the MS or non-MS group (*P* > 0.05 for each comparison) (Table 1).

### 3.2. Serum IL-6, hsCRP, FFA, and Adiponectin Levels for Uyghurs and Kazakhs in the MS and Non-MS Groups

In both the Uyghur and Kazakh populations, serum IL-6, hsCRP, and FFA levels in the MS group were higher than those in the non-MS group; overall, the levels recorded in Kazakhs were higher than those measured in Uyghurs, both with and without MS (*P* < 0.01 for each comparison). Conversely, adiponectin levels displayed the opposite trends (*P* < 0.01 for each comparison) (Table 2).

### 3.3. Detection Rates of MS and Its Components between Each Group by Quartile Assessment of Serum IL-6, hsCRP, FFA, and Adiponectin Levels in Uyghurs and Kazakhs (Q_1_ Group, Less Than the 25th Percentile; Q_2_ Group, 25th to 50th Percentile; Q_3_ Group, 50th to 75th Percentile; Q_4_ Group, Greater Than the 75th Percentile)

From the Q_1_ group to the Q_4_ group, the rates of detection for MS and its components in Uyghurs and Kazakhs tended to increase as the levels of IL-6, hsCRP, and FFA increased. Comparing the Q_4_ and Q_1_ groups, all OR values were >1, indicating that the Q_4_ group's risk was higher than that of the Q_1_ group. However, the adiponectin levels exhibited opposite results (Tables 3 [Table tab4] [Table tab5]–6).

### 3.4. Relationship of Serum IL-6, hsCRP, FFA, and Adiponectin Levels with the Number of Clustered MS Components

**Figure 1:**
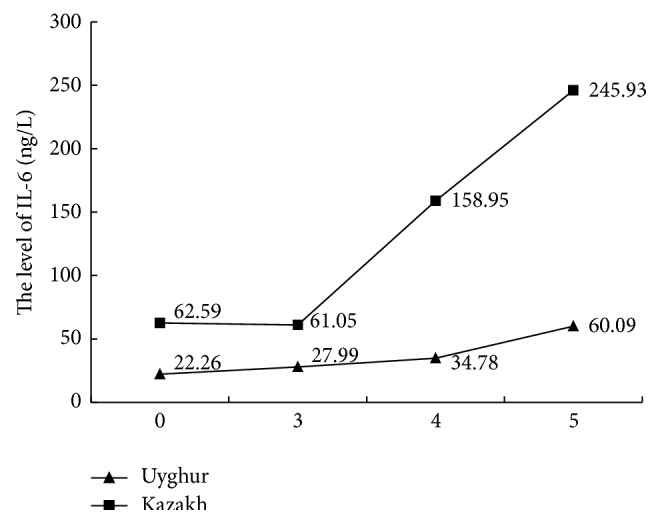
Comparing IL-6 with MS components.

**Figure 2 fig2:**
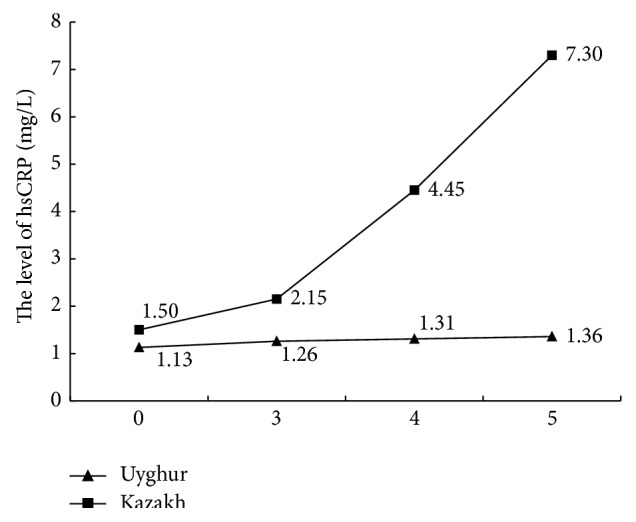
Comparing hsCRP with MS components.

**Figure 3 fig3:**
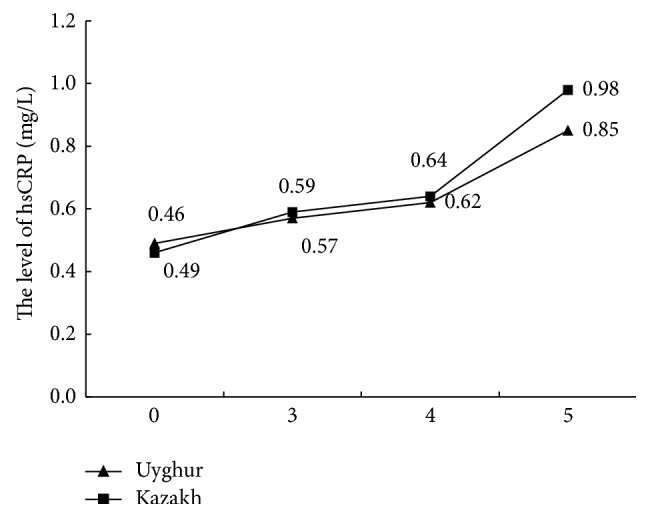
Comparing FFA with MS components.

**Figure 4:**
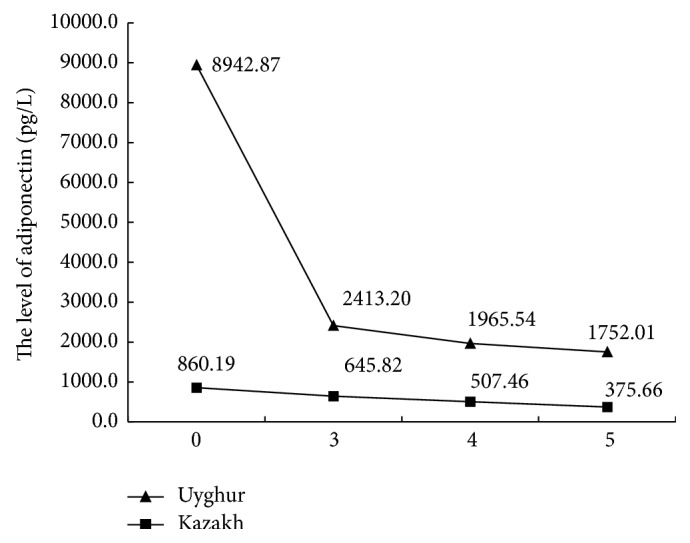
Comparing adiponectin with MS components.

As the MS component clustering increased, the serum IL-6, hsCRP, and FFA levels gradually increased in both populations (*P* < 0.01 for each comparison); however, this trend was more obvious for Kazakh individuals, as shown in the top curve. In contrast, adiponectin levels showed the opposite trend (Figures 1 [Fig fig2] [Fig fig3]–4).

## 4. Discussion

MS is a major public health problem because of its rapidly increasing prevalence and its association with type 2 diabetes and cardiovascular disease (CVD) [20]. In fact, CVD risk in patients with MS is twice that of the normal population [3]. In the United States, Europe, and India, at least 25% of adults suffer from MS. However, the reasons underling the high incidence of MS have not been confirmed. Related studies have demonstrated that MS is associated with a proinflammatory state, which is hypothesized to be related to CVD [20–23], and some inflammatory markers, such as IL-6, hsCRP, FFA, and adiponectin, are closely related to MS [21, 24, 25].

The current study analyzed the relationship between MS and inflammation in adults of rural Kazakh and Uyghur populations in northwest China. We determined that, for both populations, IL-6, hsCRP, and FFA levels were higher in the MS group than in the non-MS group and that adiponectin is an adipose tissue factor that could improve IR. This trend is similar to those reported in domestic and foreign research, though the levels in our study are higher [26–28]. Nishida et al. determined that “high” IL-6 or hsCRP levels or “low” adiponectin levels are associated with an increased risk for MS. Additionally, IL-6 and adiponectin have been shown to be important risk factors for early arterial alterations in men [29]. MS incidence was found to increase with increased CRP and IL-6 levels [30], and hsCRP levels were markedly increased in older male patients with MS [31]. Moreover, hsCRP can facilitate the prediction of new-onset CVD [32]. IL-6, hsCRP, and FFA appear to promote MS development, whereas adiponectin negatively regulates MS by suppressing IR, ultimately preventing MS.

Nishida et al. reported that individuals with an increased number of MS components have higher CRP levels [29]. In another study, when the number of components ≥ 3, FFA levels were notably increased, with a decrease in adiponectin levels [28]. The results of the present study demonstrated that IL-6, hsCRP, and FFA levels also increased among Uyghurs and Kazakhs as the clustering of MS components increased, though adiponectin levels decreased. We also analyzed the subjects according to quartiles of IL-6, hsCRP, FFA, and adiponectin levels and compared the detection rates of MS and its components from the Q_1_ group to the Q_4_ group. The results demonstrated increasing trends for the levels of IL-6, hsCRP, and FFA. Moreover, the OR values were >1 for the Q_4_ and Q_1_ groups, predicting that the incidence risk of MS and its components is higher in the Q_4_ group. However, the opposite trends were observed with increased adiponectin levels.

Uyghurs and Kazakhs are the two main ethnic groups in Xinjiang, and their unique geographical environment and living habits are very different from those of the rest of the country. For instance, their economy is primitive, their environment is harsh, their education and medical knowledge are deficient, and their self-awareness of prevention and treatment is poor. Additionally, the staple of their diet is Nang, which contains a large amount of salt, and they do not eat many fruits or vegetables. Indeed, Kazakhs consume Nang three times a day and are accustomed to drinking tea with plenty of salt and milk. They also frequently consume cured meat, which may lead to higher prevalence of MS and its components in Kazakhs compared to Uyghurs. The reported prevalence of MS in Kazakhs is 26.6% [8], whereas that in Uyghurs is 21.2% [9]. In our study, IL-6, hsCRP, and FFA levels were higher in Kazakhs than in Uyghurs, and the positive association with MS components was more obvious in the former; the results for adiponectin displayed the opposite trend. These observations provide further evidence that IL-6, hsCRP, and FFA levels are positively correlated with the occurrence of MS and that adiponectin is negatively correlated with MS.

We could not establish causal relationships in our study due to its cross-sectional design, and we did not collect data on the socioeconomic or environmental variables that could have an impact on MS. We also recruited subjects during a local investigation in northwest China and not from hospitals or other medical institutions. Subjects from hospitals or other medical institutions could be more representative, and the use of such a sample could have allowed us to explore the relationship between MS and inflammation over a range of demographic groups. Nonetheless, these findings provide important demographic insight into the growing problem of MS in rural Kazakh and Uyghur populations.

## 5. Conclusion

In conclusion, this study explored the relationship between MS and inflammation among Uyghur and Kazakh people. Despite the higher prevalence of MS in these groups compared with the rest of China, to date, few studies have investigated the underlying reasons for this higher prevalence. We observed that the abnormal expression of inflammatory cytokines might contribute to the high prevalence of MS, which could be associated with the characteristics of the different ethnicities and areas (e.g., differences in living environments, habits, and customs). To prevent MS, these groups could improve their lifestyle behaviors through education or medication to control IL-6, hsCRP, FFA, and adiponectin levels. Therefore, our observations and recommendations should be used to establish appropriate public health policies to benefit low-income populations.

## Figures and Tables

**Table 1:** Age and gender data for the Uyghur and Kazakh subjects.

Index	Uyghur		Kazakh	
	MS (*n* = 218)	Non-MS (*n* = 201)	MS (*n* = 156)	Non-MS (*n* = 180)
Sex (male/female)	104/114	95/106	74/82	88/92
Age (years)	41.49 ± 12.42	41.86 ± 11.90	42.03 ± 13.69	40.71 ± 10.24
BMI (kg/m^2^)	24.03 ± 1.73^#^	22.54 ± 2.53	27.06 ± 4.25^#^	21.76 ± 2.01
WC (cm)	92.76 ± 7.28^#^	82.73 ± 9.09	95.26 ± 10.10^#^	82.89 ± 9.32
SBP (mmHg)	140.41 ± 20.10^#^	112.95 ± 11.59	142.98 ± 22.98^#^	116.60 ± 18.24
DBP (mmHg)	93.07 ± 12.56^#^	85.24 ± 10.23	92.91 ± 13.96^#^	86.51 ± 14.79
TG (mmol/L)	1.64 ± 0.99^#^	0.86 ± 0.32	1.68 ± 1.13^#^	0.95 ± 0.37
HDL-C (mmol/L)	1.06 ± 0.25^#^	1.58 ± 0.29	1.01 ± 0.28^#^	1.54 ± 0.40
FPG (mmol/L)	4.89 ± 1.33^#^	4.49 ± 0.56	5.01 ± 1.64^#^	4.51 ± 0.95
LDL-C (mmol/L)	2.68 ± 0.72^#^	2.27 ± 0.70	2.65 ± 0.81^#^	2.25 ± 0.66
TC (mmol/L)	4.72 ± 1.08^#^	4.16 ± 0.99	4.98 ± 1.13^#^	4.20 ± 0.95

Notes: MS = metabolicsyndrome, WC = waist circumference, BMI = body mass index, SBP = systolic blood pressure, DBP = diastolic blood pressure, TG = triglyceride, TC = total cholesterol, HDL-C = high-density lipoprotein cholesterol, LDL-C = low-density lipoprotein cholesterol, and FPG = fasting plasma glucose. ^#^Comparing the index in the same ethnicity, *P* < 0.05.

**Table 2:** IL-6, hsCRP, FFA and adiponectin levels of MS and non-MS groups in the Uyghur and Kazakh populations.

	Uyghur				Kazakh			
	IL-6 (ng/L)	hsCRP (mg/L)	FFA (mg/L)	Adiponectin (pg/L)	IL-6 (ng/L)	hsCRP (mg/L)	FFA (mg/L)	Adiponectin (pg/L)
MS	30.86 (21.22, 43.89)^*∗*^	1.26 (0.79, 1.52)^*∗*^	0.57 (0.38, 0.80)^*∗*^	4405.54 (2524.4, 15964.34)^*∗*^	88.12 (44.20, 197.02)	3.20 (1.70, 5.50)	0.61 (0.48, 0.77)	446.17 (103.03, 1796.71)
Non-MS	23.32 (11.09, 31.81)^*∗*^	1.16 (0.79, 1.35)^*∗*^	0.45 (0.33, 0.65)^*∗*^	5724.58 (1824.22, 16388.89)^*∗*^	62.58 (14.20, 159.90)	1.50 (0.60, 4.50)	0.49 (0.34, 0.59)	841.71 (298.80, 2716.31)
*Z*	−5.852	−2.550	−4.398	−3.730	−3.456	−4.911	−5.518	−3.783
*P*	<0.001	0.011	<0.001	<0.001	<0.001	<0.001	<0.001	<0.001

Notes: MS = metabolic syndrome, IL-6 = interleukin-6, CRP = C-reactive protein, and FFA = free fatty acids.

^*∗*^Comparing the same index between the two ethnicities, *P* < 0.05.

**Table 3:** Detection rates of MS and components in each IL-6 level quartile.

MS and components	Uyghur						Kazakh					
	Q_1_	Q_2_	Q_3_	Q_4_	*P *	OR (Q_4_/Q_1_) (OR 95% CI)	Q_1_	Q_2_	Q_3_	Q_4_	*P *	OR (Q_4_/Q_1_) (OR 95% CI)
MS	7.6	13.4	12.9	18.4	0.000	6.27 (3.444, 11.429)	12.1	16.2	17.5	16.2	0.015	2.14 (1.128, 4.049)
Hypertension	4.5	9.8	8.6	14.6	0.000	6.28 (3.341, 11.784)	10.8	14.6	15.9	14.9	0.018	2.10 (1.113, 3.946)
Low HDL-C	19.3	19.1	18.1	22.9	0.000	10.58 (3.579, 31.261)	7.6	11.1	12.4	11.7	0.020	2.14 (1.117, 4.113)
High TG	3.6	7.2	6.4	10.3	0.000	4.16 (2.128, 8.139)	2.9	3.5	7.6	13.0	0.000	8.87 (3.892, 20.193)
High FPG	1.4	1.7	1.0	2.9	0.196	2.13 (1.768, 5.904)	4.4	7.0	8.9	9.8	0.001	3.16 (1.517, 6.570)

Notes: MS = metabolicsyndrome, TG = triglyceride, HDL-C = high-density lipoprotein cholesterol, FPG = fasting plasma glucose, OR = odds ratio, and CI = confidence interval.

**Table 4 tab4:** Detection rates of MS and components in each hsCRP level quartile.

MS and components	Uyghur						Kazakh					
	Q_1_	Q_2_	Q_3_	Q_4_	*P *	OR (Q_4_/Q_1_) (OR 95% CI)	Q_1_	Q_2_	Q_3_	Q_4_	*P *	OR (Q_4_/Q_1_) (OR 95% CI)
MS	13.1	10.7	10.5	17.7	0.015	2.17 (1.230, 3.829)	7.0	18.7	19.0	17.1	0.000	4.81 (2.428, 9.548)
Hypertension	7.4	9.5	7.9	12.6	0.008	2.43 (1.379, 4.292)	5.7	17.1	17.5	15.9	0.000	5.09 (2.532, 10.242)
Low HDL-C	17.7	18.4	19.3	24.1	0.030	3.16 (1.390, 7.184)	5.1	12.7	12.1	13.0	0.001	3.75 (1.847, 7.594)
High TG	6.0	6.2	6.0	9.3	0.047	1.89 (1.039, 3.441)	1.0	3.5	8.6	14.0	0.000	28.52 (8.280, 98.222)
High FPG	3.1	1.0	1.4	1.4	0.101	1.43 (1.057, 1.675)	2.2	8.3	8.9	9.8	0.001	6.97 (2.844, 17.079)

Notes: MS = metabolic syndrome, TG = triglyceride, HDL-C = high-density lipoprotein cholesterol, FPG = fasting plasma glucose, OR = odds ratio, and CI = confidence interval.

**Table 5 tab5:** Detection rates of MS and components in each FFA level quartile.

MS and components	Uyghur						Kazakh					
	Q_1_	Q_2_	Q_3_	Q_4_	*P *	OR (Q_4_/Q_1_) (OR 95% CI)	Q_1_	Q_2_	Q_3_	Q_4_	*P *	OR (Q_4_/Q_1_) (OR 95% CI)
MS	10.7	10.7	14.1	16.5	0.001	2.26 (1.294, 3.946)	7.4	14.0	8.9	16.1	0.003	3.44 (1.800, 6.582)
Hypertension	6.9	7.4	11.2	11.9	0.001	2.18 (1.226, 3.868)	11.3	14.6	11.0	13.7	0.926	1.15 (1.620, 2.433)
Low HDL-C	18.4	18.1	19.8	23.2	0.005	3.02 (1.364, 6.702)	6.3	9.6	6.9	11.0	0.095	1.98 (1.023, 3.825)
High TG	6.0	5.7	6.2	9.5	0.040	1.82 (1.698, 3.300)	3.0	6.3	3.3	6.0	0.305	2.00 (1.370, 4.597)
High FPG	0.7	1.7	1.9	2.6	0.041	1.34 (1.130, 2.306)	4.3	6.2	5.8	6.2	0.939	1.04 (1.437, 2.456)

Notes: MS = metabolic syndrome, TG = triglyceride, HDL-C = high-density lipoprotein cholesterol, FPG = fasting plasma glucose, OR = odds ratio, and CI = confidence interval.

**Table 6:** Detection rates of MS and components in each adiponectin level quartile.

MS and components	Uyghur						Kazakh					
	Q_1_	Q_2_	Q_3_	Q_4_	*P *	OR (Q_4_/Q_1_) (OR 95% CI)	Q_1_	Q_2_	Q_3_	Q_4_	*P *	OR (Q_4_/Q_1_) (OR 95% CI)
MS	17.9	12.4	10.7	11.0	0.000	0.31 (0.176, 0.553)	13.4	14.6	11.0	7.4	0.000	0.37 (0.195, 0.693)
Hypertension	9.5	10.0	9.1	8.8	0.323	0.88 (0.504, 1.550)	13.1	14.0	13.1	10.4	0.144	0.65 (0.353, 1.194)
Low HDL-C	24.6	18.6	16.7	19.6	0.000	0.07 (0.016, 0.302)	8.7	9.6	7.8	7.8	0.109	0.86 (0.453, 1.651)
High TG	11.2	4.8	6.2	5.3	0.001	0.33 (0.178, 0.600)	5.7	6.6	3.6	2.7	0.013	0.42 (0.176, 0.983)
High FPG	3.8	1.7	0.7	0.7	0.000	0.16 (0.046, 0.580)	5.4	8.6	5.8	2.7	0.156	0.50 (0.187, 1.334)

Notes: MS = metabolic syndrome, TG = triglyceride, HDL-C = high-density lipoprotein cholesterol, FPG = fasting plasma glucose, OR = odds ratio, and CI = confidence interval.

## Abbreviations

BMI: Body mass index
BP: Blood pressure
CI: Confidence interval
CRP: C-reactive protein
FBG: Fasting blood glucose
HDL-C: High-density lipoprotein cholesterol
HOMA-IR: Homeostasis model assessment of insulin resistance
IL-6: Interleukin-6
IR: Insulin resistance
LDL-C: Low-density lipoprotein cholesterol
MS: Metabolic syndrome
OR: Odds ratio
TC: Total cholesterol
FFA: Free fatty acids.
